# Supplementary material for: Researching New Therapeutic Approaches for Abdominal Visceral Pain Treatment: Preclinical Effects of an Assembled System of Molecules of Vegetal Origin
Source: Nutrients. 2019 Dec 20;12(1):22. doi: 10.3390/nu12010022 (PMC7019336; doi:10.3390/nu12010022)
Supplement: Supplementary file 1 [file nutrients-12-00022-s001.pdf]

Researching New Therapeutic Approaches for Abdominal Visceral Pain Treatment: Preclinical Effects of an Assembled System of Molecules of Vegetal Origin

Carmen Parisio, Elena Lucarini, Laura Micheli, Alessandra Toti, Lorenzo Di Cesare Mannelli, Giulia Antonini, Elena Panizzi, Anna Maidecchi, Emiliano Giovagnoni, Jacopo Lucci, Carla Ghelardini

Supplementary materials.

Supplementary Fig 1

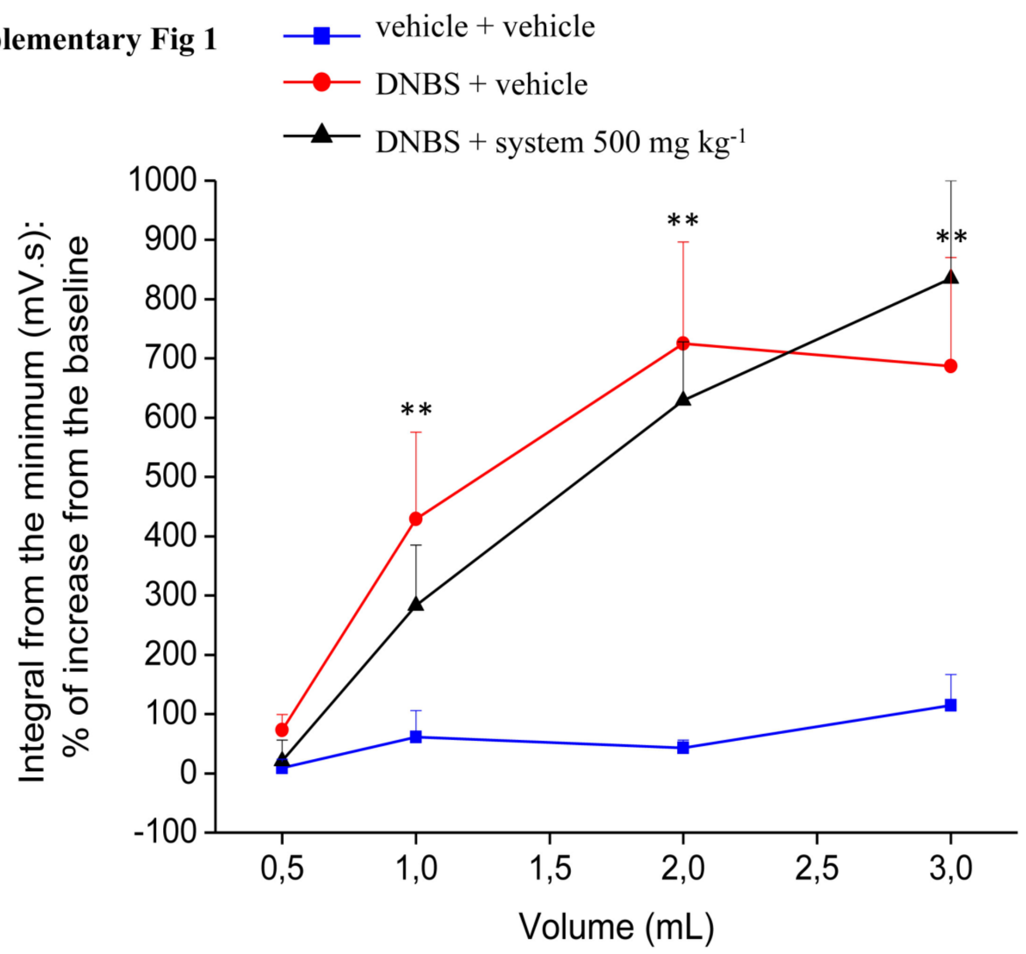

Effect of system 500 mg kg^{-1} administration on visceral hypersensitivity 3 days after DNBS injection. Visceral hypersensitivity was induced in rats by the intra-rectal injection of 2,4-dinitrobenzenesulfonic acid (DNBS, 30 mg in 0.25 mL EtOH 50%). Control animals were injected intra-rectally with saline solution. System 500 mg kg^{-1} was orally administered once daily in the animals: treatment started 3 days before DNBS injection and continued for 14 days after colitis induction. Tests were performed on day 3 after the damage induction, measuring the viscero-motor response to the colon rectal distension (0.5, 1, 2, 3 mL balloon inflation). Each value is the mean ± S.E.M. and represents the mean of 6 rat per group. **P < 0.01 vs vehicle + vehicle treated animals.

Supplementary Fig 2.a

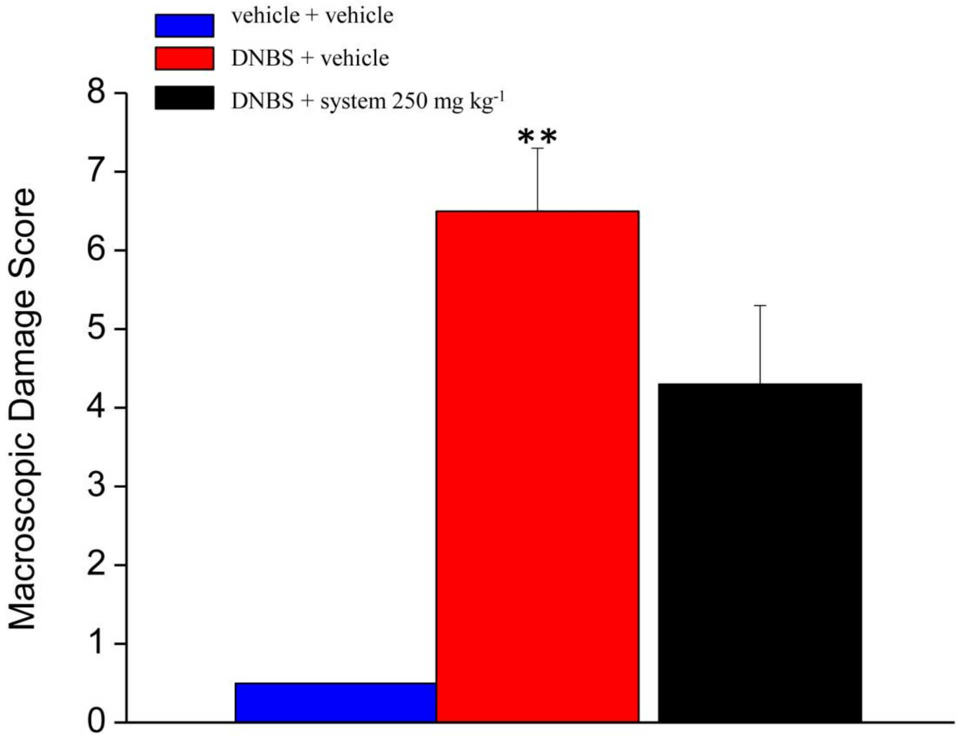

Supplementary Fig 2.b

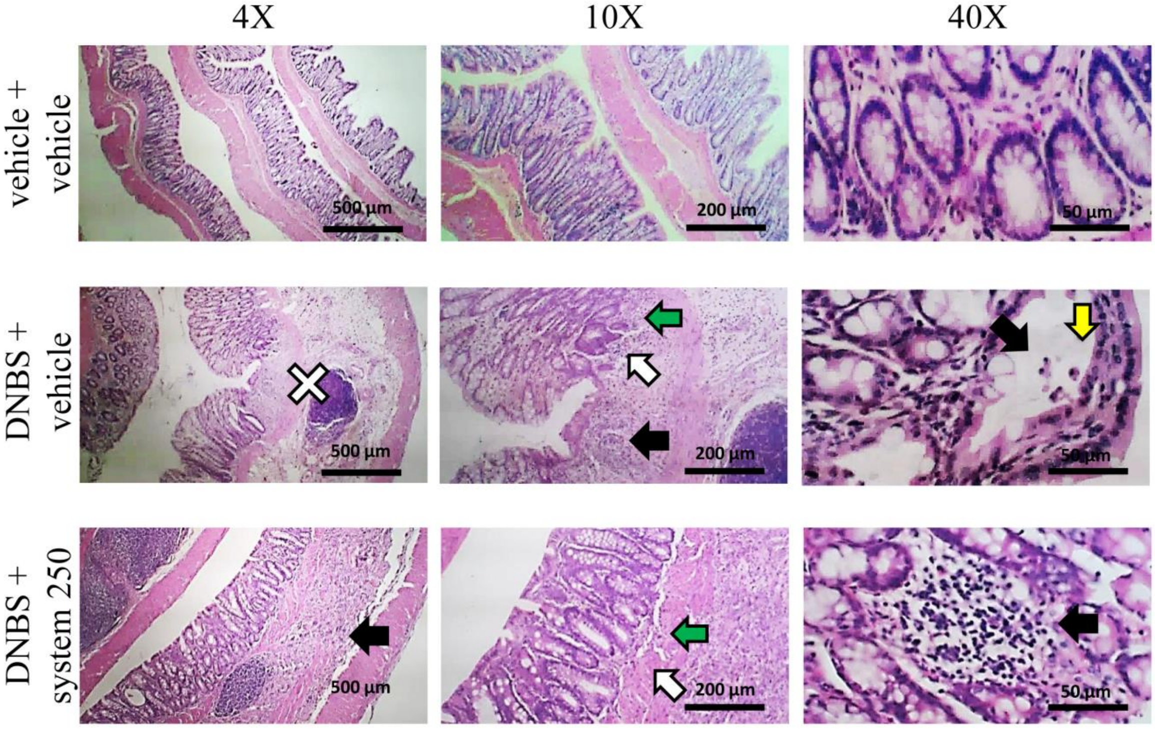

Effect of system 250 mg kg-1 administration on colon damage. Visceral hypersensitivity was induced in rats by the intra-rectal injection of 2,4-dinitrobenzenesulfonic acid (DNBS, 30 mg in 0.25 mL EtOH 50%). Control animals were injected intra-rectally with saline solution. System 250 mg kg^{-1} was orally administered once daily in the animals: treatment started 3 days before DNBS injection and continued for 14 days after colitis

induction. Animals were sacrificed 14 days after DNBS injection for both microscopic (**a**) and macroscopic (**b**) analysis. **a**) The Macroscopic Damage Score (MDS) was assigned to each animals based on: presence of adhesions between colon and other intra-abdominal organs (0-2); consistency of colonic faecal material (indirect marker of diarrhoea; 0-2); presence and extension of hyperaemia and macroscopic mucosal damage (0-5); thickening of colonic wall (mm). **b**) Microscopic evaluations were carried out by light microscopy on haematoxylin- and eosin- stained sections of colon (5 μm). In the figure it was highlighted the presence of: inflammatory mucosal cell infiltrate, submucosal and transmural, neutrophils between epithelial cells and neutrophils in crypt lumen (black arrow); granulocyte-fibrin eschar, loss of surface epithelium (white cross); goblet cell hyperplasia and mucus hypersecretion (yellow arrow); Irregular crypts, variable crypts diameters, bifurcation and branched crypts (green arrow); epithelial hyperplasia, visible as crypt elongation, with goblet cell loss (white arrow). Each value is the mean ± S.E.M. and represents the mean of 6 rat per group. **$P < 0.01$ vs vehicle + vehicle treated animals.
